# Targeted application of an organophosphate-based paint applied on windows and doors against *Anopheles coluzzii* resistant to pyrethroids under real life conditions in Vallée du Kou, Burkina Faso (West Africa)

**DOI:** 10.1186/s12936-018-2273-x

**Published:** 2018-04-02

**Authors:** Serge B. Poda, Dieudonné D. Soma, Aristide Hien, Moussa Namountougou, Olivier Gnankiné, Abdoulaye Diabaté, Florence Fournet, Thierry Baldet, Santiago Mas-Coma, Beatriz Mosqueira, Roch K. Dabiré

**Affiliations:** 1 Institut de Recherche en Sciences de la Santé (IRSS)/Centre Muraz, 01 BP 545, Bobo-Dioulasso 01, Burkina Faso; 2 Université Ouaga 1 Pr Joseph Ki-Zerbo, Ouagadougou, Burkina Faso; 3 Université Nazi Boni, Bobo-Dioulasso, Burkina Faso; 4 Institut de Recherche pour le Développement (IRD), BP 64501, 34394 Montpellier Cedex 5, France; 5 Cirad, UMR15 CMAEE, INRA, UMR1309 CMAEE, Montpellier, France; 6 Departamento de Parasitología, Facultad de Farmacia, Universitat de Valencia, Av. Vicent Andrés Estellés s/n, Burjassot, 46100 Valencia, Spain

**Keywords:** Mosquito control, Insecticide resistance, Organophosphates, Insecticide paint, Burkina Faso, Africa, Western

## Abstract

**Background:**

A novel strategy applying an organophosphate-based insecticide paint on doors and windows in combination with long-lasting insecticide-treated nets (LLINs) was tested for the control of pyrethroid-resistant malaria vectors in a village setting in Vallée du Kou, a rice-growing area west of Burkina Faso.

**Methods:**

Insecticide Paint Inesfly 5A IGR™, comprised of two organophosphates and an insect growth regulator, was applied to doors and windows and tested in combination with pyrethroid-treated LLINs. The killing effect was monitored for 5 months by early morning collections of anophelines and other culicids. The residual efficacy was evaluated monthly by WHO bioassays using *Anopheles gambiae* ‘Kisumu’ and local populations of *Anopheles coluzzii* resistant to pyrethroids. The spatial mortality efficacy (SME) at distances of 1 m was also assessed against pyrethroid-susceptible and -resistant malaria vectors. The frequency of L1014F *kdr* and *Ace*-*1*^*R*^ G119S mutations was, respectively, reported throughout the study. The Insecticide Paint Inesfly 5A IGR had been tested in past studies yielding a long-term mortality rate of 80% over 12 months against *An. coluzzii*, the local pyrethroid-resistant malaria vector. The purpose of the present study is to test if treating smaller, targeted surfaces (e.g. doors and windows) was also efficient in killing malaria vectors.

**Results:**

Treating windows and doors alone yielded a killing efficacy of 100% for 1 month against *An. coluzzii* resistant to pyrethroids, but efficacy reduced quickly afterwards. Likewise, WHO cone bioassays yielded mortalities of 80–100% for 2 months but declined to 90 and 40% 2 and 3 months after treatment, respectively. Mosquitoes exposed to insecticide paint-treated surfaces at distances of 1 m, yielded mortality rates of about 90–80% against local pyrethroids-resistant *An. coluzzii* during the first 2 months, but decreased to 30% afterwards. *Anopheles coluzzii* was reported to be exclusively the local malaria vector and resistant to pyrethroids with high L1014 *kdr* frequency.

**Conclusion:**

The combination of insecticide paint on doors and windows with LLINs yielded high mortality rates in the short term against wild pyrethroid-resistant malaria vector populations. A high SME was observed against laboratory strains of pyrethroid-resistant malaria vectors placed for 30 min at 1 m from the treated/control walls. The application of the insecticide paint on doors and windows led to high but short-lasting mortality rates. The strategy may be an option in a context where low cost, rapid responses need to be implemented in areas where malaria vectors are resistant to pyrethroids.

## Background

Malaria affects millions of people worldwide, especially in Africa. According to the latest official estimates, in 2016, there were 216 million cases of malaria worldwide compared to 237 million cases in 2010 and 211 million new cases in 2015; there were about 445,000 deaths from malaria globally, compared to 446,000 malaria deaths in 2015. Most of these deaths occurred in the African region (91%), followed by the Southeast Asian region (6%) and the Eastern Mediterranean region (2%). Children are especially vulnerable, accounting for more than two-thirds of global malaria deaths [1].

Because of the burden that malaria continues to represent, there has been a scale-up of malaria control strategies in the last 15 years. There are three main strategies: (i) vector control is mainly based on the use of insecticide-treated mosquito nets (ITNs)/long-lasting insecticide-treated nets (LLINs) and indoor residual spraying (IRS). The only class of insecticides approved for treating ITNs and LLINs are pyrethroids. IRS interventions are also mostly based on pyrethroid use, but some use dichlorodiphenyltrichloroethane (DDT) and, to a lesser extent, organophosphates (OPs) and carbamates; (ii) chemoprevention: prevention campaigns are mostly targeted at groups at risk, such as pregnant women and, more recently, children in areas where transmission is seasonal; (iii) case management: based on prompt diagnosis (such as the use of rapid diagnostic tests) and resistance management, based on artemisinin-based combination therapy (ACT).

Of these three strategies, the one showing greatest impact in reducing clinical cases of malaria in Africa is vector control and the use of ITNs and LLINs [2, 3]. Progress is being made, but the burden of malaria is still large, especially in Africa. The spread of resistance to pyrethroids due to the *kdr* mechanism could potentially hamper further progress [4–6]. There is no consensus on whether this resistance translates into reduced efficacy of malaria control programmes but there is a risk that it could [7–10]. In addition, both IRS [11] and ITNs/LLINs [12, 13] are not always accepted well by populations and/or they are used incorrectly. Because of the potential problems that widespread resistance to pyrethroids may pose and because of operational obstacles, there is a need for new tools against malaria vectors [14–16].

The present work studies the efficacy of an insecticide paint, Inesfly 5A IGR™, consisting of a microencapsulated formulation of two OPs, chlorpyrifos and diazinon, and an insect growth regulator (IGR), pyriproxyfen, all contained in a white vinyl paint with an aqueous base. Both OPs and pyriproxyfen have a different mode of action than pyrethroids, which may address the issue of resistance.

The paint may present advantages, compared to IRS, as its application does not require special equipment or trained personnel, and could lead to home improvement. Toxicology studies were performed on this product and proved its safety in terms of irritancy (ocular, dermal, systemic), cytotoxicity, mutagenicity, and allergenicity [17, 18]. It has been evaluated over a series of studies with promising results [19–22]. In particular, the latest study, the pilot pre-phase III in the VK1 village from Vallée du Kou, Burkina Faso, where the inner walls of houses were treated with the insecticide paint, in combination with LLINs [22]. Given these positive results, the present study was carried at VK3 village, also in Vallée du Kou. The objective of the present study is to test if treating a smaller surface (unlike the study carried at VK1), targeted at entry points of houses, offers protection in terms of mosquito mortality.

## Methods

### Study site

**Fig. 1:**
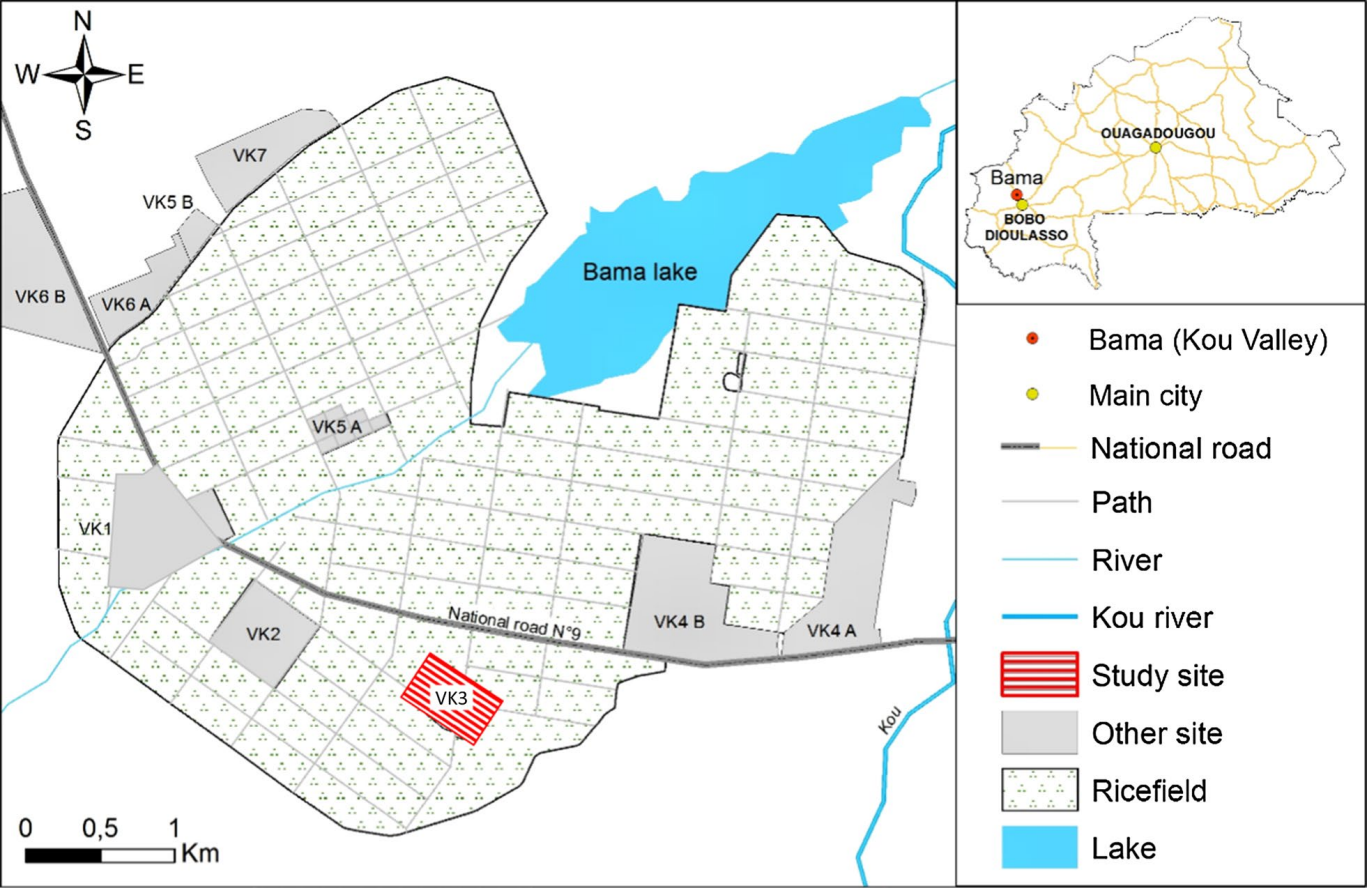
Location of VK3 at Vallée de Kou

The study was conducted in VK3 village in the Kou Valley (11° 23′ 14″N; 4° 24′ 42″W), a rice-growing area of southwestern Burkina Faso, located 30 km north of Bobo-Dioulasso (Fig. 1). Malaria vectors in the area are abundant throughout the year and the frequency of the L1014F *kdr* mutation is high, inducing high resistance levels of local malaria vectors populations to pyrethroids and DDT [23, 24]. *Anopheles coluzzii* (former *Anopheles gambiae* form M) was the only *An. gambiae* sensu lato (s.l.) species present in the area during the study, from August to December 2013, confirming previous results obtained in VK3 village during the same season. The identification of *Anopheles* species among the *An. gambiae* complex was performed using a short interspersed nuclear elements (SINE)-PCR approach [25].

### Insecticide paint and LLINs

Inesfly 5A IGR was applied on metallic doors and windows of selected houses. It was applied on two sides of doors and windows with one layer of 1 kg commercial product/6 sq m (i.e., 166.67 g/sq m). Twenty inhabited houses were selected in the village and treated with the insecticide paint. In order to assess the effectiveness of the paint + LLIN combination, each occupant of 10 treated homes received a new LLIN. These LLINs were used daily under supervision during the all survey. LLINs used during this study were new intact PermaNet 2.0, made of polyester netting impregnated with deltamethrin in a wash-resistant binder system (distributed by the National Malaria Control Programme in 2013). Ten control houses were selected and included in the study. The control houses were free of insecticide paint and new LLINs but sleepers could use any tool of their choice to protect themselves from nuisance. The 30 houses included in the study were selected according to their similarities in terms of size (the mean was 3.5 × 5 m), a distance of at least 20 m apart, with the informed consent of home owners. Houses were randomly allocated to the three arms: control, paint alone or paint + LLIN.

### Early morning collections (EMCs)

Before randomizing the treatment arms, mosquito collections took place for 1 full week in the 30 houses included in the study to ensure that there was no difference between houses in attractiveness to mosquitoes. One week after the treatment, mosquito collections were performed monthly over 4 consecutive days, between August and December 2013 (T0–T4).

Thirty volunteers from residents at VK3 of at least 18 years old were recruited for the collections. After being informed about the study, the volunteers provided written informed consent (with a fingerprint if illiterate), and received training on mosquito collection procedures. Mosquito collections were performed following World Health Organization Pesticide Evaluation Scheme (WHOPES) testing procedures [26] for mortality rates, except that inhabited houses were used rather than experimental huts. Volunteers would enter the houses at 18:00 h, one volunteer per house, and sleep under LLINs until 05:30 h. Before entering houses every evening, houses would be cleaned to eliminate scavengers (i.e., ants and other insects that might be interested in dead mosquitoes). Volunteers rotated houses each night to avoid bias in mosquito collections. At the first suspicion of malaria, volunteers were provided with curative treatment recommended by the National Malaria Control Programme in Burkina Faso. The study was approved by the Ethics Committee of the *Institut de Recherche en Sciences de la Santé* (IRSS)/*Centre Muraz*. Early in the morning, mosquito females were collected inside each house and classified as dead or alive, unfed or blood-fed. Live mosquitoes were observed for delayed mortality assessment after 24 h at 80 ± 10% relative humidity and 27 ± 2 °C temperature. All mosquitoes were then conserved in silica gel at − 20 °C to study the species and resistance status.

The design allowed the reliable assessment of the killing effect, deterrent effect and long-term residual efficacy of the treatment. Since the study was performed under real field conditions, the status (unfed or blood-fed) of female mosquitoes that entered the houses was not known, making it difficult to evaluate reliably the blood-feeding inhibition. Blood-feeding inhibition is generally assessed in experimental huts following Phase II WHOPES protocols, where the experimental design is well adapted to estimate this parameter.

### Residual efficacy tests using 30-min WHO bioassays

The World Health Organization (WHO) protocol for evaluation of residual efficacy was followed [27] using 2–3 days old unfed females of *An. gambiae* ‘Kisumu’, a reference strain susceptible to all insecticides reared at the IRSS/Centre Muraz insectarium. The local malaria vector population at VK3, identified molecularly as *An. coluzzii* and resistant to pyrethroids, was reared at the insectarium from field-collected larvae to the adult stage; it was also tested in parallel to *An. gambiae* ‘Kisumu’.

For each house, 10 females were introduced in 5 cones placed on two sides of the paint-treated surface (or control) for 30 min on metallic doors and windows, respectively. Cones were not placed on LLINs. Females were taken to the insectarium for delayed mortality assessment after 24 h at 80 ± 10% relative humidity and 27 ± 2 °C temperature. Tests were performed monthly from T0 to T4 after treatment.

### Spatial mortality assessments

The effect of mortality was also assessed at a distance. Mosquito females were placed at distances of 1 m and never came in direct contact with the treated surface. Unfed females of *An. gambiae* ‘Kisumu’ and *An. coluzzii* from VK3 raised at the insectarium from field-collected larvae were used. A total of 60 females were introduced into 4 tubes of 150 ml, with 15 females per tube. Mosquito netting was placed at both ends to allow air through. Honey-soaked cotton was introduced to ensure that females did not die from starvation. The protocol followed was the same described by Mosqueira et al. [21], except females were exposed for 30 min only, instead of 12 h. Females were taken to the insectarium for delayed mortality assessment after 24 h at 80 ± 10% relative humidity and 27 ± 2 °C temperature. Tests were performed from T0 to T4.

### Insecticide susceptibility test

The susceptibility test was performed on 2–3 days old wild female *An. gambiae* s.l. using the WHO standard vertical tube protocol [28]. Four insecticide-impregnated papers were used: permethrin 0.75%, deltamethrin 0.05%, fenitrothion 0.1%, and pirimiphos methyl 0.05%. Wild *An. gambiae* s.l. was tested against *An. gambiae* ‘Kisumu’, a fully susceptible reference laboratory strain. Mortality controls were carried out by exposing both the Kisumu strain and wild populations to non-insecticide-impregnated paper. After 1 h’s exposure, mosquitoes were transferred into insecticide-free tubes and maintained on sucrose solution. Final mortality was recorded 24 h after exposure. The threshold of susceptibility was fixed at 95% for all active ingredients used [27].

### Molecular analysis on resistance

The detection of the L1014F *kdr* mutation was performed following Martinez-Torres et al. [28] and the *ace*-*1*^*R*^ G119S mutation by Weill et al. [29] to analyse the resistance status at the time of the study. Testing took place after treatment on *An. coluzzii* females collected in 2 arms (control and treated houses) of the study houses.

### Statistical analysis

To evaluate efficacy of the insecticide paint, results were compiled and analysed using Epi Info Version 6 to test for any significant difference in mosquito entry and mortality rates between the different configurations via Chi square tests. A 95% confidence interval and a standard error were applied respectively to mortality rates and mean entries of mosquitoes in houses. When mortality rates in control houses were between 5 and 20%, Abbott’s mortality correction formula was applied [30]. Because bioassay tests are subject to variations, a 99% confidence interval was applied. The allelic frequency of each mutation (*kdr* and *ace*-*1*^*R*^) was calculated with the formula F(R) = (2RR + RS)/2n where n is the total sample size. The frequency of *kdr* and *ace*-*1*^*R*^ in *An. coluzzii* collected in the 2 arms (control and treated) of the study houses were compared by Chi square test. The genotypic frequencies at the *kdr* and *ace*-*1*^*R*^ loci were compared to Hardy–Weinberg expectations using the exact test procedures implemented in GENEPOP (version 4) software [31].

## Results

### Early morning collections (EMCs)

**Fig. 2:**
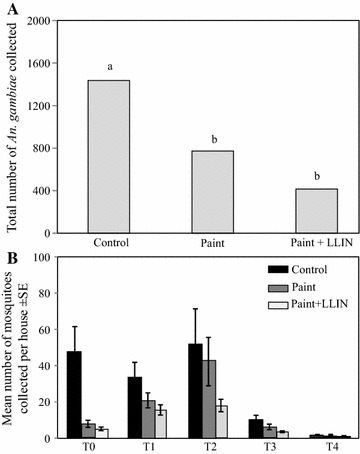
Number of *Anopheles gambiae* s.l. collected from early morning collections. **A** Total number of *Anopheles gambiae* s.l. collected per arm; **B** mean number monthly collected per house for each configuration. Letters above bars indicate significant differences between treatment arms. *LLINs* long-lasting insecticide-treated nets; *T* time in months since treatment

No difference in house attractiveness was found during the blank collections. Mosquito collections began 1 week after the treatment. The maximum mosquitoes were collected between the T1 and T2, corresponding to the peak of rainy season, and decreased to be fewer than 20 mosquitoes per house at T3 and T4, corresponding to the end of the rainy season (November–December). At the end of the experiment, overall 2624 *An. gambiae* s.l. were collected with, respectively, 1,437,772 and 415 for control, paint alone and paint + LLIN-treated houses differing significantly between control and treated houses (Fig. 2A). No repellent effect was observed between the different treatments in the months, except at T0 where there were significantly more mosquitoes collected in control houses than treated ones (Fig. 2B).

A sub-sample of 165 mosquitoes (50 ± 10 per arm for resistance gene characterization) was molecularly analysed for species identification within the *An. gambiae* complex, and were all identified as *An. coluzzii*.

Mortality rates in the houses with paint + LLIN was over 80% for 2 months, but decreased progressively, to less than 30% at T4. Mortality observed in the control houses with no paint ranged from 13 to 8% at T3 and T4 (Table 1).

**Table 1:** Mortality rates on local populations of *Anopheles coluzzii* using early morning collection

% Mortality in *Anopheles coluzzii* collected via EMCs ± CI 95%	T0	T1	T2	T3	T4
Control (LLINs and/or other methods)	0	0	0	13 ± 6.6^a^	8 ± 17.2^a^
IP/1 layer on doors and windows	100^b^	68 ± 6.4^b^	58.5 ± 4.6^b^	34 ± 12.3^b^	7.57 ± 23.2^a^
IP/1 layer on doors and windows + new LLINs	100^b^	81.1 ± 6.2^b^	60.6 ± 7.3^b^	39.4 ± 16.8^b^	28.3 ± 45.5^a^

Averages taken for each configuration, 10 houses per configuration

*IP* Insecticide paint only; *LLINs* long-lasting insecticide-treated nets; *T* time in months since treatment; *EMCs* early morning collections. Numbers in the same column sharing a letter superscript do not differ significantly (P > 0.05)

### Residual efficacy tests using 30-min WHO bioassays

The mortality rates obtained after the 30-min standard WHO cone bioassays with *An. gambiae* ‘Kisumu’ reached 100% on all treated surfaces (metallic windows and doors) during the first 3 months after paint application (T0–T2). From the painted doors (Table 2A), the residual efficacy was still superior to 80% (85%) in T3 but fell to 78% at T4. With painted windows, the mortality rates were relatively higher and rates reached 80% at T4 (Table 2A).

**Table 2:** Residual efficacy tests

% Mortality using WHO test cones ± CI 99%	*Anopheles gambiae* Kisumu (A)					*Anopheles coluzzii* VK3 (B)				
	T0	T1	T2	T3	T4	T0	T1	T2	T3	T4
Control (LLINs and/or other methods such as coils)	5 ± 2.4^a^	5 ± 2.4^a^	3 ± 1.9^a^	10 ± 3.3^a^	7 ± 2.8^a^	8 ± 3.1^a^	7 ± 2.8^a^	2 ± 1.5^a^	2 ± 1.5^a^	ND
IP/1 layer on doors + new LLINs	100^b^	100^b^	100^b^	85 ± 4.1^b^	78.5 ± 4.6^b^	100^b^	100^b^	90 ± 3.3^b^	39 ± 5.5^b^	ND
IP/1 layer on windows + new LLINs	100^b^	100^b^	100^b^	95 ± 3.1^b^	82 ± 5.3^b^	100^b^	100^b^	90 ± 4.1^b^	53 ± 6.9^b^	ND

Averages taken for each configuration, 10 houses per configuration

*IP* Insecticide Paint; *LLINs* long-lasting insecticide-treated nets; *T* time in months since treatment; *ND* not done because of insufficient numbers reared in the insectarium. Numbers in the same column sharing a letter superscript do not differ significantly (P > 0.05)

The mortality rates obtained with the local *An. coluzzii* from VK3 were between 100 and 90% from T0 to T2 and decreased significantly under 80% to reach 39 and 53%, respectively, for painted doors and windows at T3. At T4, tests were not performed with *An. coluzzii* from VK3 due to the scarcity of the local mosquito population (Table 2B).

### Spatial mortality assessments

In the control houses (windows and doors without any treatment), the mortality rates recorded were very low with less than 5% both for *An. gambiae* ‘Kisumu’ and *An. coluzzii* VK3. From T0 to T1, the distant killing effect went from 90 to 75% against *An. gambiae* ‘Kisumu’ placed for 30 min at 1 m from the painted doors and windows. At T2, this mortality decreased to 30%. By T3 and T4, mortality further decreased to 20% (Table 3A).

**Table 3:** Spatial mortality assessments on VK3 mosquitoes

% Mortality during spatial mortality assessments ± CI 99%	*Anopheles gambiae* Kisumu (A)					*Anopheles coluzzii* VK3 (B)				
	T0	T1	T2	T3	T4	T0	T1	T2	T3	T4
Control (LLINs and/or other methods)	2 ± 2.7^a^	2.9 ± 3.2^a^	1.1 ± 2.1^a^	3.6 ± 3.6^a^	3.9 ± 3.7^a^	1.7 ± 2.5^a^	2.6 ± 3.1^a^	2.9 ± 3.2^a^	2.1 ± 2.8^a^	ND
IP/1 layer on doors and windows + LLINs	90 ± 5.8^b^	75 ± 8.4^b^	30 ± 8.9^a^	20 ± 7.8^a^	20 ± 7.8^a^	80 ± 7.8^b^	77 ± 8.2^b^	25 ± 8.4^a^	15 ± 6.9^a^	ND

Averages taken for each configuration, 10 houses per configuration

*IP* Insecticide paint; *LLINs* long-lasting insecticide-treated nets; *T* time in months since treatment; *ND* not done because of insufficient numbers reared in the insectarium. Numbers in the same column sharing a letter superscript do not differ significantly (P > 0.05)

In the case of *An. coluzzii* at T0, 80% were killed. By T1, 77% of exposed individuals were killed. By T2, spatial mortality decreased to 25%, and by T3 to 15%. Tests were not performed with *An. coluzzii* at T4 for the same reason as for the residual efficacy tests given above (Table 3B).

### Resistance status and allelic frequency of the mutations *kdr L1014F* and *ace*-*1*^*R*^

**Fig. 3:**
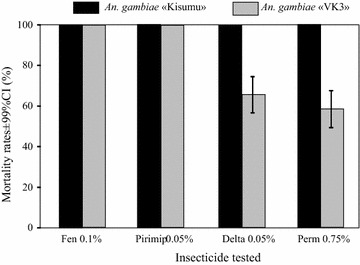
Insecticide susceptibility. Mortality rates of wild female *Anopheles coluzzii* populations and female *Anopheles gambiae* Kisumu using the WHO standard tube protocol. Fen 0.1% fenithrotion 0.1%; Pirimip 0.05% pirimiphos methyl 0.05%; Delta 0.05% deltamethrin 0.05%; Perm 0.75% permethrin 0.75%

The resistance status of wild populations of *An. coluzzii* showed that they were resistant to pyrethroids (permethrin 0.75% and deltamethrin 0.05%) with mortality rates less than 65%. They were fully susceptible to OPs (fenitrothion 0.1% and pirimiphos methyl 0.05%) with 100% of mortalities (Fig. 3). The allelic frequency of the L1014F *kdr* mutation in *An. coluzzii* females collected during EMCs at VK3 was high, averaging 94% (Table 4) without any difference between control and treated houses at the time of the study.

**Table 4:** Allelic frequency and genotype of the L1014F *kdr* and *ace*-*1*^*R*^ mutations in *Anopheles coluzzii* collected from early morning collections

Treatments	*Kdr* L1014F							*Ace*-*1*^R^ (G119S)				
	n	SS	RS	RR	*F* (L1014F)	[95% CI]	p (HW)	SS	RS	RR	*F* (G119S)	p (HW)
Control (LLINs and/or other methods)	52	1	3	48	0.952	[0.89–1.01]	0.01	52	0	0	0	–
IP/1 layer on doors and windows + LLINs	49	1	4	44	0.929	[0.86–1.00]	<0.05	49	0	0	0	–

*IP* Insecticide paint; *LLINs* long-lasting insecticide-treated nets; *EMCs* early morning collections; *n* number of mosquitoes tested; *SS* sensitive homozygote; *RS* resistant heterozygote; *RR* resistant homozygote; *F (L1014F)* allelic frequency of the *kdr* mutation; *F (G119S)* allelic frequency of the *ace*-*1*^R^ mutation; 95% CI = 95% confidence interval, *p (HW)* value for Hardy–Weinberg equilibrium hypothesis; “–” non-determinable

Within specimens analysed by PCR, no individual was detected sharing the *ace*-*1*^*R*^ mutation, as well as from mosquitoes collected in control and paint treated houses (Table 4).

## Discussion

At the VK3 village, the strategy consisted of a combination of LLINs and Inesfly 5A IGR™ applied to windows and doors. The study design reached the experimental proof concept for malaria prevention developed in “the mosquito malaria theory” [32]. The concept was recently reviewed in order to assess the hypothesis that improved housing can reduce malaria by decreasing entry of mosquitoes [33]. More particularly, a randomized controlled trial in The Gambia showed that the use of window screens and closed eaves led to a reduction in the number of mosquitoes entering houses, and a reduction in the prevalence of anaemia in children, but it did not show a reduction in malaria prevalence [34]. Other studies have shown that the closure of eaves and netting over windows can be effective in preventing mosquito entry into houses [35, 36]. However, there is little evidence that screening can reduce malaria infection.

In the VK3 study, the insecticide paint applied on windows and doors did not necessarily prevent the entry of mosquitoes, probably due to the effects of Ops, which do not have repellent characteristics [19]. However, there was a significant reduction in the numbers of mosquitoes collected in treated houses, especially into those supplied with new LLINs, compared to the control. This was probably due to the repellent effect of the deltamethrin, a pyrethroid contained in LLINs used in this study. The number of dead mosquitoes collected during EMCs was always higher in houses supplied with new LLINs, suggesting that the treatment of windows and doors combined with new LLINs yielded a high level of killing efficacy compared to control houses, but the effect lasted for only about 2 months. The Insecticide Paint Inesfly had been tested in past studies yielding a long-term mortality rate of 80% over 12 months when applied on the walls against *An. coluzzii*, the local pyrethroid-resistant population [20, 22]. The reason for this short-lasting efficacy obtained in the present study may be that the size of the treated surface was insufficient to ensure a sustained protection beyond 2 months. This is consistent with repeated observations on the importance of having a volume effect [20–22]. Another possibility is the degradation that insecticides may undergo, despite microencapsulation, when exposed to high levels of heat and sunlight [11]. This is especially the case with metallic doors and windows. The temporal efficacy of this kind of treatment could be improved beyond 3 months by increasing the treatment dose with two layers of 1 kg/6 sq m instead of one layer of 1 kg/6 sq m, or repeating the treatment of a layer of 1 kg/6 sq m 2 months later. This needs to be tested.

The mortality rates in control houses in VK3 without insecticide paint were observed in T3 and T4 only, corresponding to October and November, during which the National Malaria Control Programme (NMCP) had organized a mass distribution of new LLINS at VK3 through a universal coverage campaign. The acquisition and use of new LLINs contributed to the low mortality observed in control houses.

These results are consistent with recent studies in nearby VK1 and VK7 villages [22, 9], with mortality rates in houses with LLINs (PermaNet 2.0) only, similar to the observed rates in the present study.

Given the high killing effect of the insecticide paint against OP-susceptible mosquitoes and that this study was not carried out under controlled conditions, it was difficult to reliably assess the pyriproxyfen efficacy. Effect of the pyriproxyfen into this formulation could be assessed against OP-resistant mosquito populations in controlled conditions [19]. This allows the female mosquitoes to be sufficiently in contact with the insecticide and still live, making it possible to evaluate the effect of the IGR by monitoring their life history trait. Moreover, breeding sites close to experiment places may be polluted by the pyriproxyfen from Inesfly. The effect may be assessed on mosquito larvae.

One potential gain of the combination LLINs/paint strategy was the better management of pyrethroid-resistant mosquitoes, minimizing the risk of resistance development for OPs if used alone. Tests showed that the allelic frequency of the L1014F *kdr* mutation did not vary significantly during the testing period. This was likely because baseline frequencies are so high anyway in the area. The L1014S *kdr* mutation was not tested, but it will be monitored in future studies over a period of at least 1 year.

With regard to the *ace*-*1*^R^ mutation, *An. coluzzii* were considered to be susceptible to OPs as the distribution of the *ace*-*1*^R^ mutation is still low thus far (less than 10% overall) and in the heterozygous form. These results were consistent with the cousin study at VK1 [22]. Longer term and large data should be obtained during the Phase III study.

The killing effect at 1 m from treated surfaces confers also the possibility to be used to combat outdoor biting. Malaria transmission via outdoor biting is not negligible [37] and the potential of a longer lasting efficacy while painting larger external surfaces needs to be evaluated. A modified strategy will be tested in large scale Phase III to study the effect on malaria incidence in children in Burkina Faso but also its incidence on outdoor malaria transmission and nuisance due to other culicids. The community-based assays will include entomological and clinical assessments as well as sociological questionnaires to assess acceptability by the population. Such studies will also provide proper data on cost efficacy, which was not clearly addressed in the current study.

## Conclusions

The application of Inesfly 5A IGR™ in house-proof systems on windows and doors yielded a long-term killing efficacy of about 3 months against pyrethroid-resistant malaria vectors. *Anopheles coluzzii* in the area are highly resistant to pyrethroids but susceptible to OPs. The pilot study at VK3 showed that treating only windows and doors was not efficient in the long term. However, it may be a cost- and time-efficient tool during high transmission peaks. Painting doors and windows, or the interior of houses, should be coupled with home improvement measures to reduce the permeability of houses to vector and pest mosquitoes and other potential disease-transmitting arthropods.

New malaria control tools are needed, but they should be in line with current public health efforts and support the use of LLINs.

This strategy could be implemented for other vector-borne diseases, where vectors bite mainly outdoors, such as dengue, chikungunya and zika, as well as for nuisance caused by indoor and outdoor-biting culicids.
